# Role of Rhizospheric *Bacillus megaterium* HGS7 in Maintaining Mulberry Growth Under Extremely Abiotic Stress in Hydro-Fluctuation Belt of Three Gorges Reservoir

**DOI:** 10.3389/fpls.2022.880125

**Published:** 2022-05-27

**Authors:** Ting Ou, Meng Zhang, Yazhou Huang, Li Wang, Fei Wang, Ruolin Wang, Xiaojiao Liu, Zeyang Zhou, Jie Xie, Zhonghuai Xiang

**Affiliations:** ^1^State Key Laboratory of Silkworm Genome Biology, Key Laboratory of Sericultural Biology and Genetic Breeding in Ministry of Agriculture, College of Sericulture, Textile and Biomass Sciences, Southwest University, Chongqing, China; ^2^Kaizhou District Nature Reserve Management Center, Chongqing, China; ^3^College of Life Sciences, Chongqing Normal University, Chongqing, China

**Keywords:** hydro-fluctuation belt, rhizospheric *Bacillus megaterium*, plant growth promotion, drought stress tolerance, mechanisms

## Abstract

Plant growth-promoting rhizobacteria have been shown to play important roles in maintaining host fitness under periods of abiotic stress, and yet their effect on mulberry trees which regularly suffer drought after flooding in the hydro-fluctuation belt of the Three Gorges Reservoir Region in China remains largely uncharacterized. In the present study, 74 bacterial isolates were obtained from the rhizosphere soil of mulberry after drought stress, including 12 phosphate-solubilizing and 10 indole-3-acetic-acid-producing isolates. *Bacillus megaterium* HGS7 was selected for further study due to the abundance of traits that might benefit plants. Genomic analysis revealed that strain HGS7 possessed multiple genes that contributed to plant growth promotion, stress tolerance enhancement, and antimicrobial compound production. *B. megaterium* HGS7 consistently exhibited antagonistic activity against phytopathogens and strong tolerance to abiotic stress *in vitro*. Moreover, this strain stimulated mulberry seed germination and seedling growth. It may also induce the production of proline and antioxidant enzymes in mulberry trees to enhance drought tolerance and accelerate growth recovery after drought stress. The knowledge of the interactions between rhizobacteria HGS7 and its host plant might provide a potential strategy to enhance the drought tolerance of mulberry trees in a hydro-fluctuation belt.

## Introduction

Three Gorges Reservoir (TGR) was formed after the construction of the Three Gorges Dam in the upper reaches of the Yangtze River in China (Fan et al., 2015). The reservoir plays a major role in generating hydroelectricity and providing drinking water, in flood control, and in navigational support (Li et al., 2010). The water depth of the reservoir fluctuates greatly, ranging from 145 m (summer) to 175 m (winter), which is opposite to the natural hydrological regime in the Yangtze River. The annual 30-m variation in the water level has led to periodic flooding of large extents of the banks of the reservoir, forming a hydro-fluctuation belt (Ye et al., 2011). Artificial regulation of the water level thus leads to drought in the summer and flooding in the winter seasons. Reverse-seasonal flooding and drought have negatively impacted this habitat with vegetation degradation, soil erosion, and geological instability (Zhang M. et al., 2017). Revegetation in the hydro-fluctuation belt is considered an important eco-friendly strategy to restore the structure and function of this riparian ecosystem (Jones et al., 2009). Therefore, the utilization of drought and flooding-tolerant plant species could be a promising approach to its restoration.

Mulberry (*Morus* L.), a deciduous tree, is native in the region of the Three Gorges Reservoir and may be a good candidate for cultivation in the hydro-fluctuation belt due to the formation of a well-developed root system, strong germination, and tolerance to abiotic stress factors, such as salinity, drought, and flooding (Qin et al., 2012; Huang X. H. et al., 2013). Since it exhibits a high survival rate and recovers quickly after suffering from abiotic stress, this species has great potential for revegetation and ecosystem restoration in the hydro-fluctuation belt of the reservoir. A large variation in vigor was seen among the mulberry trees planted in 2012 at the hydro-fluctuation belt after several years of cyclical flooding and drought stress (Huang X. Z. et al., 2013). Well-growing trees were deeply rooted with strong trunks and healthy leaves, while poorly growing trees had underdeveloped root systems (Xie et al., 2021). Moreover, mulberry trees at this site often withered after suffering severe drought stress in summer (Huang X. H. et al., 2013), primarily in their initial growth stage, thus strongly interfering with their growth when planted in the hydro-fluctuation belt in TGR. Drought stress is a crucial factor that limits the establishment of mulberry trees in this area; therefore, improving the drought tolerance of mulberry may facilitate its use in revegetation efforts.

Generally, agricultural plants endued the stress tolerance features through artificial breeding approache or transgenic technology (Gupta et al., 2020). However, the improvement of mulberry drought resistance by traditional methods is restricted because of limited information on genetic variability and time consumption by woody plant species. Knowledge of the beneficial interaction between plants and root-associated microorganisms has opened new horizons for developing crops with improved drought resistance (Yang et al., 2009; Durán et al., 2018). For example, various plant growth-promoting rhizobacteria (PGPR) can improve plant productivity and increase plant resistance to abiotic or biotic stress factors (Penrose and Glick, 2003; Jahanian et al., 2012). The utilization of PGPR in crops has attracted increasing attention since it is considered eco-friendly and sustainable (Timmusk et al., 2017). In addition, some of the mechanisms responsible for plant growth promotion by PGPR have been elucidated, which include the production of phytohormones (Bashan and Holguin, 1997), inhibition of ethylene effects by the production of 1-amino-cyclopropane-1-carboxylate deaminase (Penrose and Glick, 2003), and increasing the availability of mineral nutrients (Jahanian et al., 2012). For example, the *Streptomyces* bacteria have been shown to play a core role in the drought tolerance of 30 herbaceous species and promote their growth (Fitzpatrick et al., 2018). Moreover, *Populus*, a woody plant species, recruits soil bacteria, such as Firmicutes, Acidobacteria, Chloroflexi, and Actinobacteria, to relieve the negative effects of drought (Veach et al., 2020). The application of PGPR to improve mulberry drought tolerance could therefore be a promising strategy for restoring ecosystems in the hydro-fluctuation belt.

The present study characterized the rhizobacteria associated with mulberry trees in this periodical drought region and identified one strain with a great potential to benefit the stress tolerance of mulberry. The traits involved in the antagonistic activity against phytopathogens and abiotic stress tolerance were predicted using genome sequencing methods and were further tested *in vitro*. The efficacy of this strain in alleviating drought stress of mulberry was also assessed systematically *in planta*.

## Materials and Methods

### Soil Sampling

Rhizosphere soil samples used in the study were collected from robust mulberry trees located in the hydro-fluctuation belt in Longjiao Town, Yunyang County, Chongqing Municipality, China (30°49′26″ N, 108°52′14″ E), at an elevation of approximately 172 m. Soil adhering to the surface of the root (approximately 2–5 mm) was obtained from three individual mulberry trees in September 2016 (following several months of drought stress). The primary root diameter was 2.0–2.5 cm, and the sampling depth was 15 cm below the surface. The water content of the soil was 2.8%, organic carbon was 19.2 g/kg, available phosphorus was 8.3 mg/kg, pH was 6.7, and available potassium was 134 mg/kg. The three replicated samples were transported back to the laboratory and stored at 4°C prior to further processing.

### Rhizobacterial Isolation and Identification

Rhizobacteria were isolated from the serial dilutions of rhizosphere soil. Briefly, 1 g of mixed soil was added to 100 mL of sterile distilled water to create a suspension that was then serially diluted by 10-fold. Subsequently, 100 μL suspensions of 1:1,000 to 1:100,000 dilutions were transferred into water agar (WA), Gause’s agar (GA), Luria-Bertani agar (LB), nutrient agar (NA), and potato dextrose agar (PDA) and incubated at 30°C. The cultures were observed daily and colonies with different morphological characteristics were purified as they appeared on the plates. Each isolate was cultured in potato dextrose broth (PDB) at 180 revolutions per minute (rpm) at 28°C for 16 h. Glycerol was then added to a final concentration of 50%, and the suspensions were stored at −80°C.

Molecular identification of the obtained isolates was performed as previously described by Xu et al. (2019). Genomic DNA was extracted using a PrepMan Ultra Sample Preparation Reagent kit (Applied Biosystems, Palo Alto, CA, United States), and the 16S rRNA gene was amplified using primers 27F/1492R. The amplified products were sequenced at Sangon Biotechnology Co., Ltd., Shanghai, China, and the generated sequences were analyzed against the NCBI database using BLAST to determine the sequence homology with ribosomal genes of closely related organisms. The isolates were classified at the genus level when the BLAST results indicated >97% identity.

### Screening of Plant Growth-Promoting Rhizobacteria

The ability of isolates to produce IAA and solubilize phosphate was assessed in LB (Gordon and Weber, 1951) and Pikovskaya (PVK) medium (Ames, 1964), respectively. Each purified isolate was cultured in an LB medium and a PVK medium at 28°C with three replicates. The culture suspension was then centrifuged at 12,000 rpm for 20 min. IAA activity of the supernatant was assayed daily by adding 2 mL of Salkowsky’s reagent (50 mL of 35% perchloric acid and 1 mL of 0.5 M FeCl_3_ solution) into 1 mL of supernatant and maintaining the solution in the dark for 20 min. Phosphate-solubilizing ability was assessed by adding 3 mL of ammonium vanadate-molybdate reagent (100 mL of 17.7% ammonium molybdate solution and 100 mL of 0.6% ammonium metavanadate solution with 33.3% nitric acid) into 1 mL of supernatant. The optical density (OD) of the prepared solutions was measured at 530 and 490 nm to determine the content of IAA and phosphate, respectively.

### Genome Sequencing and Identification of HGS7

HGS7 strain was selected for further examination based on the plant growth-promoting traits exhibited *in vitro*. Genomic DNA was extracted using PrepMan Ultra Sample Preparation Reagent (Applied Biosystems, United States) according to the manufacturer’s instructions. The genome was sequenced using a Pacific Biosciences RSII (PacBio, Menlo Park, CA, United States) platform. *De novo* assembly of the genome from PacBio sequence reads was performed using the hierarchical genome-assembly process (HGAP) software. GeneMark software was used to predict the open reading frames in the genome sequence. tRNAs and rRNAs were predicted using tRNAscan and rRNAmmer software, respectively. Gene functions were annotated using a variety of function-related databases, including the Non-Redundant (Nr) protein database, the Swissprot database, the Pfam database, and the Clusters of Orthologous Groups (COG) protein database. A circular chromosomal genome map with COG functional annotations was plotted using Circos v0.62, and secondary metabolite synthesis gene clusters were predicted using antiSMASH 4.0. The general features and sequences of other representative *B. megaterium* strains were downloaded from the NCBI database.

The morphological characteristics of HGS7 grown on LB plates at 28°C for 24 h were recorded. Gram staining and spore staining were performed as described by Benson (2002) and observed under an optical microscope. A series of biochemical tests, including Voges-Proskauer and starch hydrolysis tests, were conducted using an HK-MID-66 kit (HUANKAI, China) following the protocol provided by the manufacturer. Phylogenetic trees of HGS7 based on 16S rRNA gene sequences and the single-copy nuclear genes of 15 bacterial strains related to the *Bacillus* genus were constructed by applying the neighbor-joining method using MEGA version 6.0 with 1,000 replicates of bootstrap values (Tamura et al., 2011).

### Antagonistic Activity to Phytopathogens and Abiotic Stress Tolerance of HGS7

The antagonistic activity of the HGS7 strain against several plant pathogens was evaluated *in vitro*. HGS7 cells were cultured in modified PDB (MPDB: 200 g of potato, 20 g of maltose, 10 g of peptone, 5 g of (NH_4_)_2_SO_4_, and 1.5 g of Na_2_HPO_4_ per liter) at 28°C for 3 days at 180 rpm. Cell-free supernatant was obtained by passing it through a 0.22-μm micropore filter that was then introduced into a sterile PDA medium (10%, *v*/*v*). Fresh plugs of pathogens (5 mm diameter) grown in PDA were inoculated onto a PDA containing cell-free supernatant and incubated at 25°C. Pathogens cultured on PDA plates without supernatant served as controls. Each treatment was carried out in triplicate. The diameter of each fungal colony was determined from the average of measurements made in three different orientations, and the level of inhibition was calculated as follows: percent inhibition = (control colony diameter – treated colony diameter)/control colony diameter × 100%.

The abiotic stress tolerance of HGS7 was assessed *in vitro*. Freshly cultured HGS7 cells were inoculated in LB broth amended with different concentrations of NaCl (0, 2, 4, 6, 8, 10, and 12%; *w*/*v*) and then incubated at 28°C at 180 rpm. HGS7 strain was also cultured in LB in which pH was adjusted with 1 M HCl and 1 M NaOH to obtain a pH range of 3.0–10.0 (Rajnish et al., 2015) and incubated at 28°C at 180 rpm. Finally, the HGS7 strain was cultured in LB at 180 rpm at different temperatures (8, 15, 22, 29, 36, 43, 50, and 57°C). Growth was determined in all cases by measuring the optical density of cultures at 600 nm after 24 h.

### Effect of HGS7 on the Growth and Stress Tolerance of Mulberry

HGS7 cells were cultured in King’s B medium for 18 h at 28°C at 180 rpm. The fresh culture was centrifuged at 5,000 rpm for 10 min and resuspended in sterilized distilled water to obtain final concentrations of 1.0 × 10^5^, 1.0 × 10^6^, 1.0 × 10^7^, and 1.0 × 10^8^ colony forming units per milliliter (CFU/mL).

Healthy mulberry seeds (“Guisangyou 62”) were disinfected and germinated as described by Xie et al. (2017). The surface-disinfected mulberry seeds were immersed in bacterial suspensions of different concentrations for 24 h. Seeds soaked in sterilized distilled water were used as a control. Each treatment comprised five replicates of 30 seeds that were placed in a 9-cm-diameter petri dish containing sterilized moistened filter paper and maintained at 25°C with a photoperiod of 12 h at 200 μmol⋅m^–2^⋅s^–1^ and 70% humidity. The germination potential implying the vitality of seeds was determined 4 days after inoculation. The seed germination rate and radicle/plumule length of the germinated seeds were calculated after 15 days.

Surface-sterilized seeds were sown in plastic pots (12 × 12 cm^2^ diameter) containing 300 g of sterilized soil and cultivated at 25°C with a photoperiod of 12 h at 200 μmol⋅m^–2^⋅s^–1^ and 70% humidity. At the two-leaf stage, 30 mL of bacterial suspensions of different concentrations or sterile water alone were applied to each pot. Five independent replicates were used for each treatment. Fifty days after the application of HGS7 suspension to the soil, the root/shoot length, fresh weight (FW), and dry weight (DW) of three randomly selected individual seedlings were measured.

Polyethylene glycol 6000 (PEG) was used to simulate drought stress (Caruso et al., 2008) and was used at concentrations of 5, 10, 15, and 20% PEG (*w*/*v*) to yield −0.035, −0.166, −0.325, and −0.534 MPa water potential, respectively. As mentioned earlier, 90 days after inoculation of suspension of HGS7 (1.0 × 10^7^ CFU/mL) onto mulberry seedlings, 30 mL of PEG of different concentrations or sterilized water alone were applied to each pot. Mulberry trees were cultivated at 25°C with a photoperiod of 12 h at 200 μmol⋅m^–2^⋅s^–1^ and 70% humidity. Each treatment was replicated 10 times. Then, 7 days after the treatment with PEG, four seedlings were randomly collected and immediately frozen in liquid nitrogen. Proline levels and the activity of antioxidant enzymes, including superoxide dismutase (SOD), catalase (CAT), and peroxidase (POD), were then analyzed using biochemical kits (Suzhou Comin Biotechnology Co., Ltd., China) following the manufacturer’s instructions. The remaining seedlings were irrigated with sterilized water to evaluate the recovery potential. Fifty milliliters of water were initially applied to each pot to flush out the PEG from the soil. Thereafter, 30 mL of sterilized water was added to each pot every 3 days. Shoot and root length and fresh and dry weight of four randomly selected mulberry seedlings from each treatment were calculated 105 days after the initiation of recovery.

### Statistical Analysis

Data analyses were carried out using SPSS (version 17.0). Differences between treatments in the seed germination, seedling growth, and the effect of HGS7 on mulberry drought tolerance were analyzed using Tukey’s one-way analysis of variance (ANOVA). ***, **, and * indicated significant differences at *P* < 0.001, *P* < 0.01, and *P* < 0.05, respectively.

## Results

### Characterization of Rhizobacterial Isolates

A total of 74 isolates were obtained in September after drought stress from rhizosphere soil of robust mulberry growing in the hydro-fluctuation belt of the Three Gorges Reservoir. The bacteria were classified into four phyla and 16 genera (Table 1) based on their near full-length 16S rRNA gene sequences which were deposited in the GenBank under accession numbers MK602381-MK602394, MK602519-MK602577, and MK583533. The predominant phylum was Proteobacteria (48 of the 74 isolates, 64.9%), followed by Firmicutes (19 of the 74 isolates, 25.7%). Pseudomonadales (60.4%), Enterobacteriales (31.3%), and Burkholderiales (8.3%) were the most frequently detected taxonomic orders within the 48 Proteobacterial isolates, while only the order Bacillales was detected within Firmicutes. The dominant genera in the collection of 74 isolates were *Pseudomonas* (32.4%), *Bacillus* (25.7%), and *Klebsiella* (12.2%). Members of *Acinetobacter*, belonging to the family Moraxellaceae, also accounted for 5.4% of the total culturable bacterial population. The relative abundance of the remaining genera was less than 5%, with single isolates of *Rugamonas*, *Enterobacter*, and *Leclercia*.

**TABLE 1:** Taxonomy information of rhizospheric bacteria of mulberry trees.

Phyla	Classes	Orders	Families	Genera	Number of isolates	Relative abundance (%)
Proteobacteria (48)	Gammaproteobacteria	Pseudomonadales	Pseudomonadaceae	*Pseudomonas* spp.	24	32.4%
				*Rugamonas* sp.	1	1.4%
			Moraxellaceae	*Acinetobacter* spp.	4	5.4%
		Enterobacteriales	Enterobacteriaceae	*Klebsiella* spp.	9	12.2%
				*Lelliottia* spp.	2	4.1%
				*Enterobacter* sp.	1	2.7%
			Erwiniaceae	*Pantoea* spp.	3	1.4%
	Betaproteobacteria	Burkholderiales	Burkholderiaceae	*Paraburkholderia* spp.	2	2.7%
				*Burkholderia* sp.	1	1.4%
			Oxalobacteraceae	*Massilia* sp.	1	1.4%
Firmicutes (19)	Bacilli	Bacillales	Bacillaceae	*Bacillus* spp.	19	25.7%
Actinobacteria (3)	Actinobacteria	Streptomycetales	Streptomycetaceae	*Kitasatospora* spp.	2	2.7%
		Micrococcales	Micrococcaceae	*Arthrobacter* sp.	1	1.4%
Bacteroidetes (4)	Flavobacteriia	Flavobacteriales	Flavobacteriaceae	*Flavobacterium* sp.	1	1.4%
				*Chryseobacterium* spp.	2	2.7%
			Oxalobacteraceae	*Massilia* sp.	1	1.4%

### Determination of Plant Growth Promotion Potential of Isolates

**FIGURE 1:**
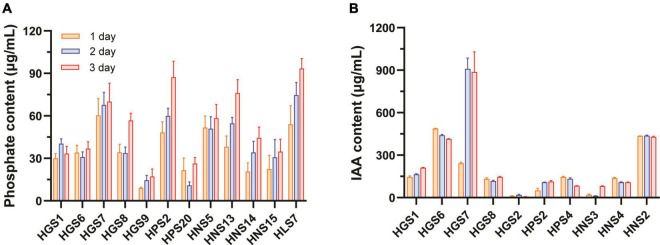
Phosphate-solubilizing and IAA-producing capacity of rhizospheric bacteria of mulberry trees. **(A)** Phosphate-solubilizing activity. **(B)** IAA-producing activity. Data represent mean ± standard deviation (*n* = 3).

A total of 12 isolates showed P solubilization (Figure 1A). After 1 day of growth, *Bacillus* sp. HGS7, *Pseudomonas* sp. HNS5, and *Acinetobacter* sp. HLS7 solubilized more phosphate of 60.4, 51.7, and 54.1 μg/mL, respectively. While slower, *Bacillus* sp. HPS2 and *Lelliottia* sp. HNS13 also solubilized substantial phosphorous (over 50 μg/mL) after 2 days of incubation. Overall, *Bacillus* sp. HGS7, *Bacillus* sp. HPS2, *Lelliottia* sp. HNS13, and *Acinetobacter* sp. HLS7 exhibited the greatest phosphorous-solubilizing capacity, yielding soluble phosphate concentrations of 70, 87.4, 76.1, and 93.4 μg/mL after 3 days of incubation, respectively.

A total of 10 out of the 74 isolates produced IAA (Figure 1B), with *Klebsiella* sp. HGS6 and *Pantoea* sp. HNS2 accumulating IAA at concentrations of 487.4 and 435.5 μg/mL by 1 day of incubation, respectively. The production of IAA by *Klebsiella* sp. HGS6 and *Pantoea* sp. HNS2, however, did not increase with time. In contrast, IAA accumulation in *Bacillus* sp. HGS7 significantly increased over time, measuring 909.7 and 887.7 μg/mL by days 2 and 3, respectively.

*Bacillus* sp. HGS7 was screened for more extensive analyses due to its prominent traits previously associated with plant growth promotion, such as IAA production and phosphate-solubilizing activity. A quantitative analysis of phosphate concentration released in cultured HGS7 cells revealed concentrations of 70 μg/mL by day 3, after which the concentration gradually declined (Supplementary Figure 1A). This strain also produced high amounts of IAA, reaching concentrations of 242.8 μg/mL by day 1 and 1,073.6 μg/mL by day 4 (Supplementary Figure 1B).

### Identification and Genomic Analyses of HGS7

**FIGURE 2:**
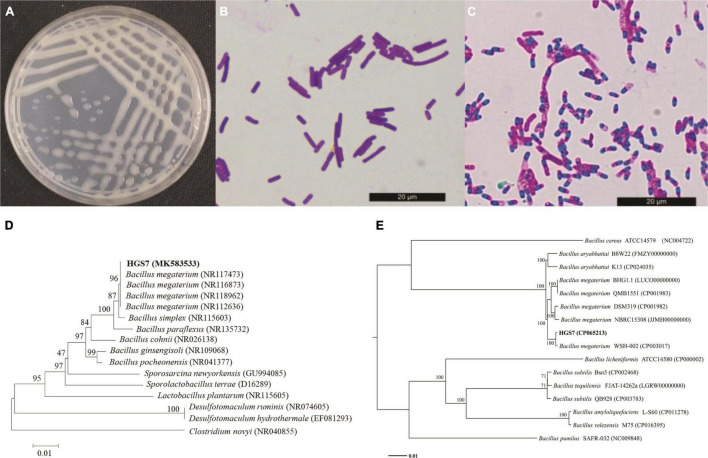
Characterization of *B. megaterium* HGS7. **(A)** Colony features on LB after 24 h. **(B)** Gram-positive staining. **(C)** Endospore staining. **(D)** Phylogenetic tree based on 16S rRNA genes. **(E)** Phylogenetic tree based on single-copy nuclear genes. The trees were constructed by MEGA version 6.0 using neighbor-joining analyses of 1,000 bootstrap replications.

The HGS7 strain forms white colonies on LB plates (Figure 2A) and is a Gram-positive (Figure 2B), spore-forming (Figure 2C), and rod-shaped bacterium. Biochemical assays indicated that HGS7 was negative for the Voges-Proskauer reaction and starch hydrolysis but positive for the utilization of sucrose and lactose (Supplementary Table 1). The phylogenetic analysis of the sequence of the full-length 16S rRNA gene of HGS7 indicated that it clustered most closely to *Bacillus megaterium* and in the same minimal clade as *B. megaterium* (NR117473) (Figure 2D). A phylogenetic tree based on the single-copy nuclear genes revealed that HGS7 was most closely related to *B. megaterium* (CP003017) (Figure 2E). Therefore, the HGS7 strain was identified as *Bacillus megaterium* based on its morphological, biochemical, and molecular characteristics.

**FIGURE 3:**
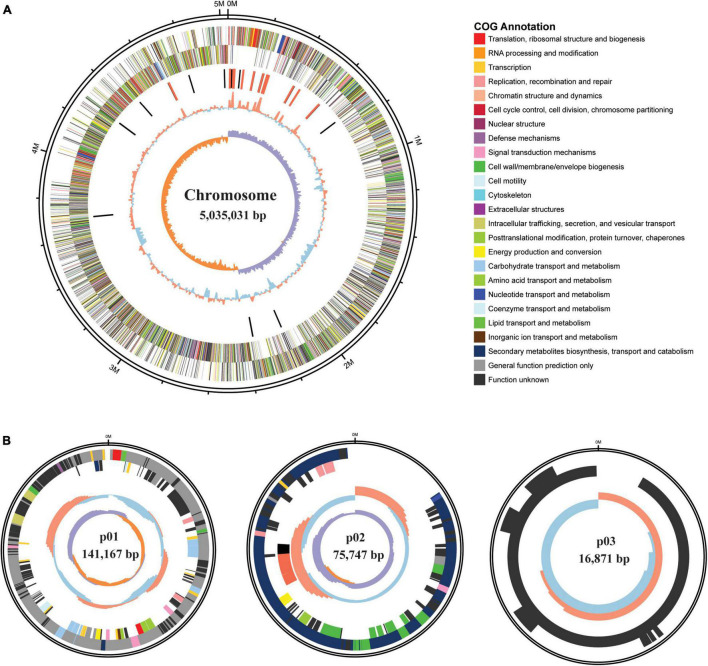
Genome maps of *B. megaterium* HGS7. **(A)** Circular chromosome. **(B)** Plasmids. From outside of the map to inside: size of the genome; predicted protein-coding genes on the positive strand (circle 2) and negative strand (circle 3) with different colors representing different COG functional classifications; rRNA (red) and tRNA (black); GC content, with > 38.27% GC (red) and ≤ 38.27% GC (blue); GC skew, with G% > C% in purple and G% < C% in orange.

The complete circular genome of HGS7 comprises 5.03 Mb, and this strain also has three circular plasmids: p01 (141,167 bp), p02 (75,747 bp), and p03 (16,871 bp) (Table 2 and Figure 3). The G + C contents of the chromosome and three plasmids are 38.27, 33.99, 36.17, and 34.74%, respectively. The chromosome possesses 5,214 predicted coding sequences (CDSs) with an average length of 806 bp. Among the predicted genes, 1,913 (36.7%) could be classified into COG families composed of 21 categories (Supplementary Table 2). Annotations were assigned to 5,169 (99.14%) putative genes based on similarity searches within the Nr database, while 45 (0.86%) CDSs were predicted to encode proteins with unknown or hypothetical functions (Table 2). The general features of *B. megaterium* HGS7 genome and other representative *B. megaterium* strains are summarized in Supplementary Table 3. Moreover, the bioinformatic analysis showed that the HGS7 genome contained seven putative gene clusters for the biosynthesis of secondary metabolites using antiSMASH, which included three terpenes, a phosphonate, a type III polyketide synthase (T3pk), a lasso peptide, and a siderophore (Supplementary Table 4).

**TABLE 2:** General features of the *B. megaterium* HGS7 genome.

Feature	Chromosome	Plasmid 1	Plasmid 2	Plasmid 3
Size (bp)	5,035,031	141,167	75,747	16,871
G + C content (%)	38.27	33.99	36.17	34.74
Number of CDSs	5,214	163	71	20
Average CDS length (bp)	806	671	754	581
tRNA	112	0	17	0
rRNA	39	0	3	0
Number of genes with assigned function	5169 (99.14%)	71 (43.56%)	35 (49.30%)	2 (10.00%)
Number of genes without assigned function	45 (0.86%)	92 (56.44%)	36 (50.70%)	18 (90.00%)

*CDSs represent protein-coding sequences.*

The analysis of the whole annotated genome sequence revealed that HGS7 contained several genes that likely contribute to plant growth promotion and stress tolerance enhancement. Genes involved in the synthesis of amidohydrolase and aldehyde dehydrogenase were identified, which could convert indole-3-acetamide and indole-3-acetaldehyde to IAA (Table 3 and Supplementary Table 5; Shao et al., 2015; Liu et al., 2016). A potential phosphate solubilization pathway was also detected, wherein *gdhII* and *Hhipl*2 encode a glucose dehydrogenase, which could catalyze glucose to gluconic acid. This product could solubilize mineral phosphates (Gupta et al., 2012). Additionally, *rhbC, yfmD*, and *hemH* genes were involved in the biosynthesis of ferrochelatase and siderophore ABC transporter permease. Other genes (*alsS, alsD, bdhA*, and *aco*) enable the production of volatile organic compounds (VOCs) (Table 3 and Supplementary Table 5). Moreover, HGS7 contained genes that enable the metabolism of antibiotics, such as chalcone (*pks11*), bacitracin (*bcr, bce, nprM*), and thiazole (*GLX3, thiF*). In addition to the plant growth-promoting and antifungal traits, *B. megaterium* HGS7 also contained several genes that encoded for metabolites contributing to abiotic stress tolerance (Table 3 and Supplementary Table 5). For example, the *tre* gene cluster encoded trehalose production, a compound that functions as an osmoprotectant (Duan et al., 2013). The genes *ytlC*, *spe*, and *pot* involved in the production of spermidine, which are capable of scavenging free radicals, were also annotated in the genome (Zhang H. W. et al., 2017). Additionally, several genes responsible for the production of cold/heat/alkaline-shock proteins and oxidative stress enzymes, such as superoxide dismutase (*sod*) (Wu et al., 2016), catalase (*kat*) (Shin et al., 2008), and peroxidase (*PRXQ, per, ykuU*) (Su et al., 2018), were also identified in the genome, all of which potentially protect bacterial cells from exogenous oxidative stress.

**TABLE 3:** Potentially beneficial genes of *B. megaterium* HGS7 for plant growth.

Gene	Gene annotation	Description	References
*yhcX^a^*, *nfdA*, *Aldh2*, *aldHT*, *ald*, *ywdH*, *gbsA*	Amidohydrolase and aldehyde dehydrogenase	IAA synthesis	Shao et al., 2015; Liu et al., 2016
*ykoQ*, *yidA*, *xpaC*, *gdhII^b^*, *Hhipl2*, *ypgQ*	Phosphatase, phosphohydrolase, glucose dehydrogenase	Phosphate solubilization	Gupta et al., 2014
*rhbC*, *yfmD^c^*, *hemH*	Siderophore biosynthesis protein	Siderophore production	Baysse et al., 2001; Saxena et al., 2020; da Silveira et al., 2020
*alsS*, *aco*, *acu*, *ybdG*, *bdhA*	Acetoin and 2,3-butanediol synthesis	Volatile organic compounds	Hernández-Calderón et al., 2018; Huo et al., 2018
*pks11*	Chalcone	Antimicrobial compounds	Waddell et al., 2005
*bcr*, *bce*, *nprM*	Bacitracin	Antimicrobial compounds	Eva et al., 2008
*GLX3*, *thiF*	Thiazole	Antimicrobial compounds	Xi et al., 2001; Lehmann et al., 2006
*treA^b^*, *treR*, *treP*	Trehalose	Osmoprotectant	Duan et al., 2013
*opuAA*, *opuAB*, *opuAC*	Glycine/betaine	Osmoprotectant	van der Heide and Poolman, 2000
*speE1*, *speE2*, *speG*, *potA-D*, *ytlC*	Spermidine	Osmoprotectant	Zhang H. W. et al., 2017
*PRXQ*, *perR*, *ykuU*	Peroxidase	Stress response protein	Su et al., 2018
*katX*, *katE*, *katA*	Catalase	Stress response protein	Shin et al., 2008
*sodC1*, *sodC2*, *sodF*, *sodA*	Superoxide dismutase	Stress response protein	Wu et al., 2016
*ureA-I*	Urease	Stress response protein	Zhou et al., 2016
*speA*	Lysine decarboxylase	Stress response protein	Liu et al., 2016
*panD*	Aspartate 1-decarboxylase	Stress response protein	Liu et al., 2016

*Genes were annotated based on Non-redundant (Nr) protein database (a: COG database; b: Swissprot database; c: Pfam database).*

### Antagonistic Activity to Phytopathogens and Abiotic Stress Tolerance of HGS7

Given that the HGS7 strain contained multiple genes encoding antimicrobial substances, its antagonistic effect on the growth of various phytopathogens was assessed *in vitro*. Several plant pathogens were inhibited by the cell-free supernatant of HGS7 (Supplementary Figure 2). It strongly inhibited the hyphal growth of *Sclerotinia sclerotiorum* and *Scleromitrula shiraiana*, causal agents of mulberry fruit disease (Table 4). Cell-free supernatants of HGS7 also inhibited *Fusarium oxysporum*, *Fusarium solani*, and *Phoma exigua*, but to a little extent.

**TABLE 4:** Inhibitory activities of cell-free supernatant of *B. megaterium* HGS7 on phytopathogens.

Pathogen	Growth inhibition (%)
*Sclerotinia sclerotiorum* PZ-2*^a^*	83.8 ± 10.0
*Scleromitrula shiraiana* SXSG-5*^b^*	64.1 ± 7.1
*Fusarium solani* SWU12	28.3 ± 2.1
*Fusarium oxysporum* SWU24	21.8 ± 0.8
*Cochliobolus sativus* SWU25	39.0 ± 5.6
*Botrytis cinerea* SWU5	56.1 ± 3.4
*Alternaria alternata* SWU26	35.3 ± 7.0
*Phoma exigua* GXH1*^c^*	29.4 ± 11.1
*Beauveria bassiana* SWU40	40.6 ± 15.6
*Ceratocystis ulmi* SWU10	32.2 ± 1.8

*Growth inhibition was calculated as the percentage of growth relative to that of the untreated test organism after 3 days of inoculation (a: 1-day, b: 9-day; c: 7-day). Data represent mean ± standard deviation (n = 3).*

**FIGURE 4:**
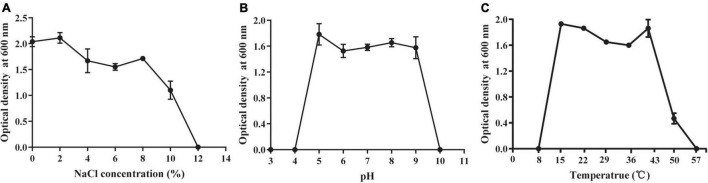
Growth of *B. megaterium* HGS7 under different cultural conditions. **(A)** Different concentrations of NaCl. **(B)** Different pH values. **(C)** Different temperatures. Data represent mean ± standard deviation (*n* = 3).

The abiotic tolerance of HGS7 was assessed to determine whether it had the ability to grow in stressful environments. This strain exhibited exceptional tolerance to salt in the media containing up to 8% NaCl and even showed limited growth in 10% NaCl (Figure 4A). Moreover, HGS7 could grow well in media with pH ranging from 5.0 to 9.0 (Figure 4B) and at temperatures from 15 to 50°C, although the growth rate was less at temperatures above 43°C (Figure 4C).

### Growth Promotion and Drought Stress Alleviation of Mulberry by HGS7

Given that many genes are involved in the biosynthesis of potential plant growth-promoting and stress mitigation substances, the effects of HGS7 on the growth and survival of mulberry seedlings were evaluated.

**FIGURE 5:**
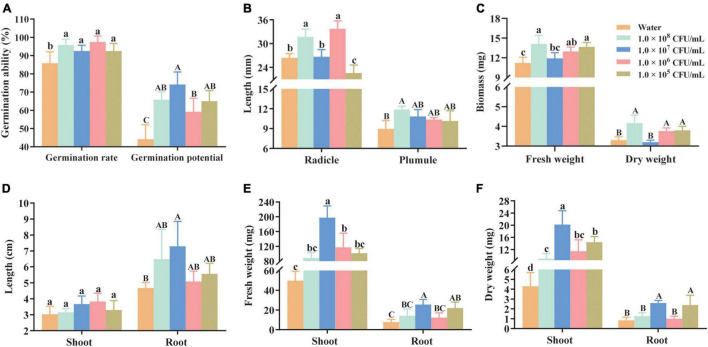
Effects of *B. megaterium* HGS7 on the germination of mulberry seeds and seedling growth. **(A)** Germination rate and potential of mulberry seeds. **(B)** Radicle and plumule length from germinated mulberry seeds. **(C)** Fresh and dry weight of germinated mulberry seeds. **(D)** Shoot and root length of mulberry seedlings. **(E)** Fresh weight of root and shoot of mulberry seedlings. **(F)** Dry weight of shoot and root of mulberry seedlings. Data represent mean ± standard deviation (*n* = 3). Different letters indicate treatments that exhibited significant differences at *P* < 0.05.

The immersion of seeds in a cell suspension of HGS7 at various concentrations significantly (*P* < 0.05) increased the rate of seed germination (Figure 5A). Seedling growth was also enhanced after inoculation with cells at different concentrations (Figures 5B,C) compared to control plants. The greatest germination potential was observed when the seeds were immersed in a 1.0 × 10^7^ CFU/mL suspension of HGS7 (Figure 5A). The concentration of 1.0 × 10^6^CFU/mL suspension also significantly (*P* < 0.01) increased the radicle length by 21.67% (from 26.42 to 33.73 mm) (Figure 5B). An increase in the germination potential/rate and biomass (FW and DW) was also detected when mulberry seeds were immersed in a 1.0 × 10^5^ CFU/mL bacterial suspension of HGS7 (Figures 5A,C). However, the radicle length was lower than that of the control at this concentration of HGS7 (Figure 5B).

The application of HGS7 cell suspension to mulberry seedlings also resulted in an increase in the root and shoot growth relative to water control (Figure 5 and Supplementary Figure 3). The highest growth promotion was observed in 1.0 × 10^7^ CFU/mL HGS7 treatment, where length, fresh weight, and dry weight of roots increased by 56.11, 229.88, and 213.25%, respectively (Figures 5D–F). This treatment also enhanced the fresh and dry weights of shoots by 297.29 and 369.77%, respectively (*P* < 0.001) (Figures 5E,F). Moreover, the 1.0 × 10^6^ CFU/mL HGS7 treatment facilitated seedling growth, with the dry and fresh weight of shoots increasing by 166.74 and 135.95%, respectively (Figures 5E,F).

**FIGURE 6:**
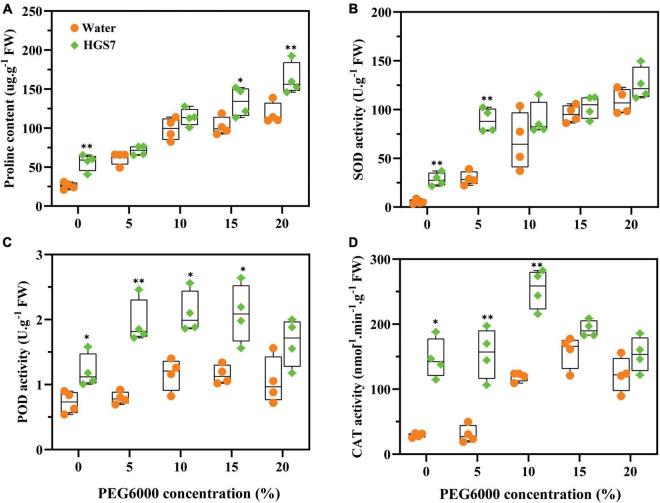
Effects of *B. megaterium* HGS7 on anti-stress substrates of mulberry. **(A)** Proline. **(B)** SOD. **(C)** POD. **(D)** CAT. Data represent mean ± standard deviation (*n* = 4), **P < 0.01, *P < 0.05.

The capability of HGS7 to enhance drought stress tolerance in mulberry was assessed by analyzing the biochemical properties of mulberry leaves after inoculating the plants. Although the concentration of proline increased with escalating drought stress even in control plants, seedlings inoculated with HGS7 had significantly higher proline concentrations than control plants in the presence of the highest PEG concentrations (Figure 6A). The largest increase in proline concentration (51%) was seen in plants treated with HGS7 that were not exposed to PEG, while it was 15 and 41% higher after the application of 15 and 20% PEG, respectively (Figure 6A). Moreover, HGS7-inoculated mulberry also exhibited higher reactive oxygen species (ROS) scavenging capacity, as indicated by the elevated levels of antioxidant enzyme activity. Specifically, SOD activity was significantly (*P* < 0.01) higher in HGS7-inoculated mulberry than in control mulberry treated with 5% PEG, where SOD increased from 29.5 to 89.1 U/g (Figure 6B). Additionally, HGS7 significantly (*P* < 0.05) enhanced mulberry POD activity, which increased by 59.39, 44.76, 44.90, and 36.29% compared to that of control plants treated with 0, 5, 10, and 15% PEG, respectively (Figure 6C). CAT activity was also significantly (*P* < 0.01) higher in HGS7-inoculated plants than in controls, increasing by 80.20 and 53.01% in plants treated with 5 and 10% PEG (Figure 6D).

**FIGURE 7:**
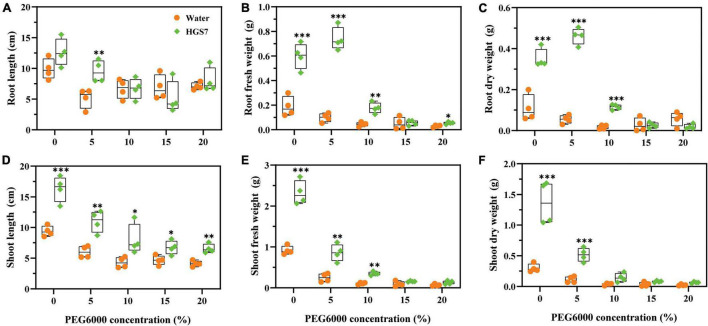
Effects of *B. megaterium* HGS7 on mulberry recovery after suffering from drought stress. **(A)** Root length. **(B)** Root fresh weight. **(C)** Root dry weight. **(D)** Shoot length. **(E)** Shoot fresh weight. **(F)** Shoot dry weight. Data represent mean ± standard deviation (*n* = 4), ***P < 0.001, **P < 0.01, *P < 0.05.

Seven days after drought stress, mulberry trees were irrigated with sterile water to test their ability to recover from drought stress. Mulberry seedlings inoculated with HGS7 recovered from drought stress, conferred by higher concentrations of added PEG, and were much better than control plants. About 105 days after the end of imposition of drought stress, in the groups applied with 5% PEG, mulberry treated with HGS7 had 45.7% longer roots than control plants (Figure 7A). Moreover, the application of HGS7 enhanced the biomass of mulberry roots compared to the controls. The fresh weight of HGS7-treated plants was increased (45.7, 83.0, and 74.7%) (Figure 7B), and their dry weight was also increased (84.6, 87.8, and 85.2%) in plants treated with 0, 5, and 10% PEG, respectively (Figure 7C). In addition, mulberry seedlings treated with HGS7 had longer shoots than controls, that is, 42.5, 45.1, 46.3, 30.9, and 37.01% (*P* < 0.05) longer in plants treated with PEG, respectively (Figure 7D). The shoot biomass of the HGS7-inoculated plants treated with 0, 5, 10, 15, and 20% PEG was also significantly higher (Figures 7E,F).

## Discussion

The growth of mulberry trees in the hydro-fluctuation belt of the Three Gorges Reservoir Region was restricted by drought stress, which impeded its revegetation capacity (Xie et al., 2021). The growing evidence has demonstrated that the symbioses of *Bacillus* species common in stressful habitats with plants can mitigate the negative effects of drought stress by enhancing the plant response to such environmental perturbations (Walia et al., 2014; Zhou et al., 2016; Ullah et al., 2019; de Vries et al., 2020). In the present study, the application of *B. megaterium* strain HGS7, which is capable of producing IAA and solubilizing phosphate, had beneficial effects on mulberry seedling growth (Figure 5) and its drought tolerance by inducing proline and antioxidant enzymes in plants (Figure 6). Such traits are critical to maintaining osmotic pressure and reducing oxidative stress injury in plant cells (Gururani et al., 2012; Tiwari et al., 2016). Thus, *B. megaterium* strain HGS7 might be exploited to relieve the adverse effects of drought stress and improve crop yields in crops.

Apart from promoting growth in plants suffering from abiotic stress, *Bacillus* strains could confer resistance in host plants against biotic stresses by several mechanisms, such as producing antimicrobial compounds, competing for nutrient resources, and modulating plant resistance responses (Saxena et al., 2020; WoldemariamYohannes et al., 2020; Poveda and González-Andrés, 2021). *Bacillus* strains have served as biological agents to manage diseases that negatively affect agricultural crops (Li et al., 2015). The synthesis of various antibiotics, such as chalcone, phenazine, bacitracin, and thiazole, is essential for their efficacy against phytopathogens (Gupta et al., 2014; McRose and Newman, 2021). For example, *B. cereus* YN917 contains gene clusters responsible for the biosynthesis of phenazine and thus serves as a biocontrol agent against rice blast (Hu et al., 2021). Interestingly, many genes contributing to the production of these antibiotics were annotated in the genome of *B. megaterium* HGS7, and this strain exhibited substantial extraordinary inhibition of several pathogens *in vitro* (Table 4). These results suggest that *B. megaterium* HGS7 could be useful for disease control in other crops.

*Bacillus megaterium*, a large group of the *Bacillus* sp., was named due to its large cell size (Wang et al., 2016), which is ubiquitous in various environments, especially in the soil (Vary et al., 2007). A series of studies focus not only on its beneficial effects in plants (Eppinger et al., 2011; Wang et al., 2020) but also on its application as a probiotic in the feed industry, due to its biosafety for host and adaptability to a wide range of temperature (0–45°C) (Vos et al., 2009). For example, *B. megaterium* SG183 strain has been shown to exhibit no toxicity *in vitro* (hemolysis, cytotoxicity, antibiotic resistance, and genotoxicity) and *in vivo* (acute toxicity) assessments, and it showed potential as a dietary supplement (Kotowicz et al., 2019). Non-pathogenicity to the host plant or animal should be a critical precondition during the probiotic selection (Verschuere et al., 2000). Although *B. megaterium* HGS7 strain serves as a good candidate biofertilizer for the mulberry trees in the hydro-fluctuation belt of the TGR and *B. megaterium* is often reported as a probiotic, its biosafety should be further evaluated in the model animals such as silkworm, mouse, and fish.

To explore the beneficial aspects of HGS7, it is essential to analyze and compare their genetic information with typical *B. megaterium*. Based on complete genome sequences, different *B. megaterium* isolates carry diverse chromosomal genes, which might be the reason why they exhibit complex biological functions (Eppinger et al., 2011). Most strains of *B. megaterium* carry multiple plasmids, usually more than four (Vary et al., 2007). Both *B. megaterium* QMB1551 (Eppinger et al., 2011) and *B. megaterium* 1259 (Deng et al., 2021) have seven endogenous plasmids, and *B. megaterium* H2 strain has 18 circular plasmids (Oyewusi et al., 2021). Our genomic analysis showed that *B. megaterium* HGS7 contained three circular plasmids, which are less than QMB1551 and more than DSM319 (Supplementary Table 3). The genome size of microbes is positively correlated with their adaptability to different environments (Konstantinidis and Tiedje, 2004). One typical characteristic of soil microorganisms is the presence of a high number of non-coding RNAs, which is helpful for fast growth, successful sporulation, germination, and rapid response to changes in the availability of nutrients (Klappenbach et al., 2000; Shrestha et al., 2007; Yano et al., 2013; Andres-Barrao et al., 2017). Genomic features indicated that the HGS7 strain possessed a higher number of tRNAs, which might facilitate its adaptation to various environments. Furthermore, the genome sequence of the HGS7 strain might provide evidence for several potential mechanisms that are responsible for the manifestation of its effects on mulberry development and enhancement of drought tolerance. HGS7 strain contains several genes that are involved in the metabolism of hormones and the production of volatile organic compounds (Table 3 and Supplementary Table 5). The metabolites like IAA, phosphatase, and acetoin could stimulate plant growth, in particular, the growth of roots (Ryu et al., 2003). Wang et al. (2020) also found that *B. megaterium* NCT-2 strain, which contains 10 indigenous plasmids and 53 rRNAs that play a role in the bioremediation of secondary salinization soil, possesses genes involved in the synthesis of auxins and alkaline phosphatase. Additionally, HGS7 contains genes encoding for spermidine and trehalose production, as well as for the production of antioxidant enzymes, which function as osmoprotectants and regulators of growth in plants under environmental stress (Duan et al., 2013). *B. megaterium* BOFC15, a spermidine-producing rhizobacterial strain, improved drought resistance of *Arabidopsis* (Zhou et al., 2016). Our findings indicated that the HGS7 strain conferred greater drought tolerance to mulberry trees. The presence of these genes confers the ability to defend against oxidative stress, thus contributing to microbial colonization and survival required for improved plant growth (Adeleke et al., 2021). The presently isolated HGS7 strain exhibited extraordinary capability to endure various pH, temperature, and salt conditions *in vitro*, thus suggesting the potential ability to stimulate plant growth in adverse conditions. This phenomenon is also observed in the bacteria of other genera, such as *Klebsiella* sp. (Liu et al., 2014) and *Paenibacillus* sp. (Xie et al., 2016), both of which contain functional genes that enable the production of anti-stress metabolites and enhance plant growth in unfavorable conditions. While the whole genome sequence of HGS7 enables us to catalog its many potentially beneficial traits, further studies are needed to identify those that contribute to its modulation of plant fitness.

**FIGURE 8:**
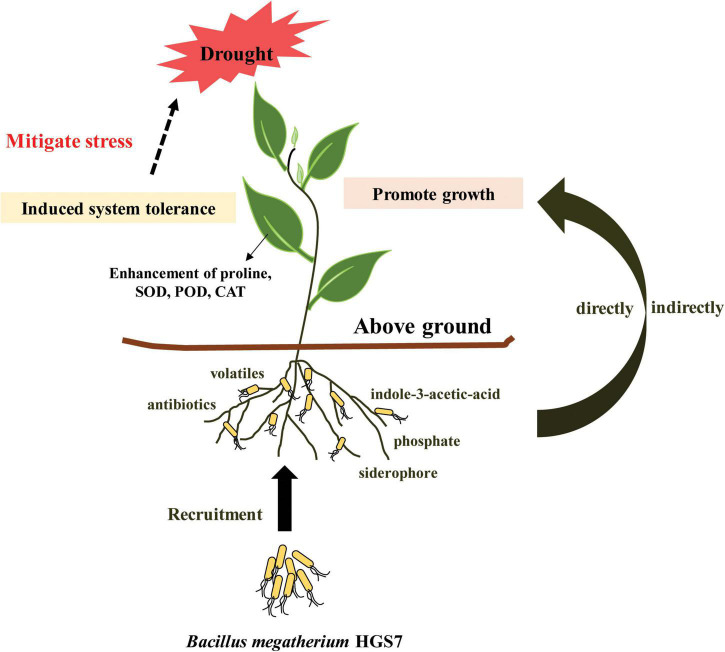
A proposed model of *B. megaterium* HGS7-mediated drought tolerance mechanisms in mulberry.

Based on our findings, a model of a mechanistic pathway for HGS7-mediated drought tolerance in mulberry is proposed, which could benefit the plant through both direct and indirect mechanisms (Figure 8). The secretion of exogenous IAA, production of volatiles and siderophores, and solubilization of phosphate might stimulate mulberry growth. Induced systemic tolerance pathways could also enhance drought tolerance, such as by triggering the accumulation of anti-stress enzymes or osmotic substances. Furthermore, HGS7 can protect the plant against pathogens by producing antibiotics, volatiles, and siderophores, which indirectly contribute to mulberry growth. Thus, *B. megaterium* strain HGS7 might enhance the resistance of mulberry trees to waterlogging stress after drought and maintain mulberry growth under extremely abiotic stress conditions in the hydro-fluctuation belt of Three Gorges Reservoir, which requires further investigation and confirmation. The current study sheds light on understanding the genomic basis of using *B. megaterium* HGS7 to promote mulberry growth and enhance its tolerance in response to drought stress and offers a possible approach to increase drought tolerance of mulberry in the hydro-fluctuation belt. Meanwhile, it is necessary to uncover the mechanism of *B. megaterium* HGS7-mediated drought stress alleviation in mulberry at the molecular level in the future through transcriptome analysis, gene expression, knockout, and complementation experiments.

## Conclusion

The vigor of mulberry trees planted in the Three Gorges Reservoir Region was greatly restricted by the drought stress in summer. *B. megaterium* HGS7, isolated from the mulberry rhizosphere of the hydro-fluctuation belt in the present study, possessed multiple functional genes that are involved in PGP traits, that is, IAA production and phosphate solubilization. The inoculation of HGS7 facilitated mulberry growth in a greenhouse and also enhanced its tolerance to drought stress. The beneficial effects of HGS7 on mulberry growth provide a promising strategy to overcome the drought pressure in the hydro-fluctuation belt. The application of plant-beneficial microorganisms not only increases crop productivity in agriculture but also provides an eco-friendly strategy for the bioremediation field.

## Data Availability Statement

The datasets presented in this study can be found in online repositories. The names of the repository/repositories and accession number(s) can be found below: https://www.ncbi.nlm.nih.gov/genbank/, MK602381–MK602394, MK602519–MK602577, MK583533, and CP065213–CP065216.

## Author Contributions

TO and JX designed the experiments. TO, MZ, YH, LW, FW, and RW performed the experiments. TO analyzed and wrote the manuscript. JX and XL revised the manuscript. ZZ and ZX conceived the study and contributed resources. All authors contributed to the article and approved the submitted version.

## Conflict of Interest

The authors declare that the research was conducted in the absence of any commercial or financial relationships that could be construed as a potential conflict of interest.

## Publisher’s Note

All claims expressed in this article are solely those of the authors and do not necessarily represent those of their affiliated organizations, or those of the publisher, the editors and the reviewers. Any product that may be evaluated in this article, or claim that may be made by its manufacturer, is not guaranteed or endorsed by the publisher.
