# Supplementary material for: Role of Rhizospheric Bacillus megaterium HGS7 in Maintaining Mulberry Growth Under Extremely Abiotic Stress in Hydro-Fluctuation Belt of Three Gorges Reservoir
Source: Front Plant Sci. 2022 May 27;13:880125. doi: 10.3389/fpls.2022.880125 (PMC9195505; doi:10.3389/fpls.2022.880125)
Supplement: Supplementary file 1 [file Data_Sheet_1.docx]

Supplementary Material

**TABLE S1**│ Physiological and biochemical characteristics of the HGS7 strain.

| Tests items | Results | Tests items | Results |
| --- | --- | --- | --- |
| Sucrose | + | Lactose | + |
| Voges Proskauer | ‒ | Urease | ‒ |
| Glucose | + | Glycerin fermentation | ‒ |
| 3% H_2_O_2_ | + | H_2_S formation | ‒ |
| Nitrate | + | Trisaccharide iron | + |
| Maltose | + | Semi-solid agar | + |
| Mannose | + | Starch | ‒ |
| Peptone water | ‒ | Simon's citric acid | + |

Note: ‘+’ represents positive (growth or reaction) and ‘–’ represents negative (no growth or no reaction).

**TABLE S2│**Functional categories of *B. megaterium* HGS7 genome according to the COG database.

| Type | Functional categories | COG |
| --- | --- | --- |
| Information storage and processing | Chromatin structure and dynamics | 1 |
|  | Transcription | 92 |
|  | Translation, ribosomal structure and biogenesis | 132 |
|  | Replication, recombination and repair | 93 |
| Cellular processes and signaling | Cell cycle control, cell division, chromosome partitioning | 22 |
|  | Cell motility | 40 |
|  | Cell wall/membrane/envelope biogenesis | 81 |
|  | Posttranslational modification, protein turnover, chaperones | 65 |
|  | Signal transduction mechanisms | 57 |
|  | Intracellular trafficking, secretion, and vesicular transport | 19 |
|  | Defense mechanisms | 26 |
| Metabolism | Energy production and conversion | 108 |
|  | Coenzyme transport and metabolism | 106 |
|  | Secondary metabolites biosynthesis, transport and catabolism | 24 |
|  | Lipid transport and metabolism | 54 |
|  | Nucleotide transport and metabolism | 63 |
|  | Inorganic ion transport and metabolism | 114 |
|  | Amino acid transport and metabolism | 177 |
|  | Carbohydrate transport and metabolism | 116 |
| Poorly characterized | Function unknown | 285 |
|  | General function prediction only | 239 |
| Total |  | 1913 |

**TABLE S3│** Genomic feature comparison of *B. megaterium* HGS7 and representative *Bacillus* *megaterium* strains.

| Features | *B. megaterium* HGS7 | *B. megaterium* DSM319 | *B. megaterium* QMB1551 | *B. megaterium* SGAir0080 | *B. megaterium* 1259 |
| --- | --- | --- | --- | --- | --- |
| Genome size (Mb) | 5.03 | 5.09 | 5.09 | 5.07 | 6.41 |
| G + C content (%) | 38.27 | 38.2 | 38.2 | 38.2 | 37.5 |
| Plasmid | 3 | — | 7 | 8 | 18 |
| tRNA | 112 | 115 | 120 | 35 | 47 |
| rRNA | 39 | 11 | 11 | 130 | 149 |
| Total predicted CDS | 5214 | 5124 | 5130 | 5339 | 6962 |

‘–’ represents none of plasmids in strains and CDS represents protein-coding sequences.

**TABLE S4│**Identification of gene clusters potentially involved in secondary metabolite synthesis in the *B. megaterium* HGS7 genome.

| Cluster | Position | Types | Metabolites | Functions |
| --- | --- | --- | --- | --- |
| 1 | 618137-638985 | Terpene | Carotenoid | Antioxidant |
| 2 | 747824-789341 | Phosphonate | Unknown | Unknown |
| 3 | 1435906-1476991 | T3pks | Unknown | Unknown |
| 4 | 2077358-2099235 | Terpene | Unknown | Unknown |
| 5 | 2102862 -2149767 | Lasso peptide | Paeninodin | Antimicrobial |
| 6 | 3634603-3670382 | Terpene | Surfactin | Antimicrobial |
| 7 | 3822921 -3839460 | Siderophore | Unknown | Accumulate and take up iron |

**TABLE S5│** Genes in *B. megaterium* HGS7 associated with plant growth promotion properties.

| Description | Gene | Gene ID | COG ID | Gene annotation |
| --- | --- | --- | --- | --- |
| IAA synthesis | *yhcX*^a^ | 464 | COG0388 | amidohydrolase |
|  | *nfdA* | 4746 | COG1574 | amidohydrolase |
|  | *Aldh2* | 1902 | COG1012 | aldehyde dehydrogenase |
|  | *ald* | 1505 | COG1012 | aldehyde dehydrogenase |
|  | *ywdH* | 2017 | COG1012 | aldehyde dehydrogenase |
|  | *gbsA* | 2177 | COG1012 | betaine aldehyde dehydrogenase |
|  | *aldHT* | 2295 | COG1012 | aldehyde dehydrogenase |
| Phosphate uptake and solubilization | *ykoQ* | 4301 | COG1408 | phosphoesterase |
|  | *yidA* | 592 | COG0561 | phosphatase |
|  | *xpaC* | 1890 | COG4915 | phosphatase |
|  | *LPPD* | 3961 | COG0671 | phosphatase |
|  | *cpdA* | 3905 | — | phosphatase |
|  | *gdhII*^b^ | 3342 | COG1028 | glucose dehydrogenase |
|  | *Hhipl2* | 1660 | — | glucose dehydrogenase |
|  | *ypgQ* | 1849 | COG1418 | phosphohydrolase |
|  | *Mesh1* | 2341 | COG0317 | phosphohydrolase |
|  | *phoB* | 1158 | COG1785 | alkaline phosphatase |
|  | *phoP* | 2865 | COG0745 | alkaline phosphatase |
|  | *ykoX* | 384 | COG0586 | alkaline phosphatase |
|  | *hssR* | 1989 | COG0745 | alkaline phosphatase |
|  | *ykaA* | 4653 | COG1392 | phosphate transport regulator |
|  | *phoU* | 4461 | COG0704 | phosphate transport regulator |
|  | *pstB* | 4462 | COG1117 | phosphate transport regulator |
|  | *pstB1* | 741 | COG1117 | phosphate ABC transporter |
|  | *pit*^c^ | 4652 | COG0306 | inorganic phosphate transporter |
| Siderophore production | *rhbE*^a^ | 3999 | COG3486 | siderophore biosynthesis protein |
|  | *rhbC* | 4001 | COG4264 | siderophore biosynthesis protein |
|  | *rhbF*^a^ | 3997 | COG4264 | siderophore biosynthesis protein |
|  | *yfiZ* | 4940 | COG0609 | siderophore ABC transporter permease |
|  | *yfmD* | 806, 1431 | COG0609 | siderophore ABC transporter permease |
|  | *hemH* | 537 | COG0276 | ferrochelatase 2 |
| Acetoin and 2,3-butanediol synthesis | *alsD* | 710 | COG3527 | alpha-acetolactate decarboxylase |
|  | *alsS* | 711 | COG0028 | acetolactate synthase |
|  | *acoA*^a^ | 1769 | COG1071 | acetoin dehydrogenase E1 |
|  | *acoB*^a^ | 1770 | COG0022 | acetoin dehydrogenase E1 |
|  | *acoC*^a^ | 1771 | COG0508 | acetoin dehydrogenase E2 |
|  | *acoR*^b^ | 1773 | COG3284 | acetoin operon transcriptional activator |
|  | *acuA* | 4759 | — | acetyltransferase (GNAT family) |
|  | *acuB* | 4760 | COG0517 | acetoin utilization protein |
|  | *acuC* | 4761 | COG0123 | acetoin utilization protein |
|  | *ybdG* | 2732 | COG0596 | acetoin dehydrogenase E2 subunit |
|  | *bdhA* | 1774 | COG1063 | 2,3-butanediol dehydrogenase |
| Chalcone | *pks11* | 1399 | COG3424 | chalcone synthase |
| Imidazolonepropionase | *hutI* | 3097, 1555 | COG1228 | imidazolonepropionase |
|  | *hisF* | 4994 | COG0107 | imidazole glycerol phosphate synthase cyclase |
|  | *hisH* | 4996 | COG0118 | imidazole glycerol phosphate synthase |
|  | *hisB* | 4997 | COG0131 | imidazole glycerol phosphate dehydratase |
| Bacitracin | *bcrA* | 2000 | COG1131 | bacitracin ABC transporter ATP-binding protein |
|  | *bceA* | 3293, 3797 | COG1136 | bacitracin ABC efflux transporter ATP-binding protein |
|  | *bceB* | 3294 | COG0577 | bacitracin export permease protein |
|  | *nprM* | 2242 | COG3227 | bacillolysin |
| Thiazole | *GLX3* | 2949 | COG0693 | thiazole biosynthesis protein ThiJ |
|  | *thiF* | 4963 | COG0476 | thiazole biosynthesis adenylyltransferase ThiF |
| Trehalose | *treA*^b^ | 887 | COG0366 | glycoside hydrolase |
|  | *treR* | 888 | COG2188 | trehalose operon repressor |
|  | *treP* | 886 | COG1263 | trehalose permease IIC protein |
| Glycine/betaine | *opuAA* | 1481 | COG4175 | glycine/betaine ABC transporter |
|  | *opuAB* | 1482 | COG4176 | glycine/betaine ABC transporter |
|  | *opuAC* | 1483 | COG2113 | glycine/betaine ABC transporter |
| Spermidine | *speE1* | 701, 3569, 5123 | COG0421 | spermidine synthase |
|  | *speE2* | 2859 | COG4262 | spermidine synthase |
|  | *speG* | 3653 | COG1670 | spermidine N1-acetyltransferase |
|  | *potA* | 166 | COG3842 | spermidine/putrescine ABC transporter |
|  | *potB* | 167 | COG1176 | spermidine/putrescine ABC transporter |
|  | *potC* | 168 | COG1177 | spermidine/putrescine ABC transporter |
|  | *potD* | 169 | COG0687 | spermidine/putrescine ABC transporter |
|  | *ytlC* | 4812 | COG1116 | spermidine/putrescine ABC transporter |
| POD | *PRXQ* | 366 | COG1225 | peroxiredoxin |
|  | *perR* | 367 | COG0735 | peroxide operon regulator |
|  | *ykuU* | 921 | COG0450 | peroxiredoxin YkuU |
| SOD | *sodC1* | 2012 | COG2032 | superoxide dismutase |
|  | *sodC2* | 4877 | COG2032 | superoxide dismutase |
|  | *sodF* | 2672 | COG0605 | superoxide dismutase |
|  | *sodA* | 4465 | COG0605 | superoxide dismutase |
| CAT | *katX* | 2984 | COG0753 | catalase |
|  | *katE* | 3870 | COG0753 | catalase |
|  | *katA* | 5172 | COG0753 | catalase |
| Stress response protein | *ysnF* | 412 | NO | stress response protein |
|  | *yflT* | 686 | No | stress response protein |
|  | *csbD* | 942 | COG3237 | stress response protein |
|  | *yxiE* | 1796 | COG0589 | stress response protein |
|  | *yceC* | 2648 | COG2310 | stress response protein |
|  | *ysnF* | 4590 | COG3861 | stress response protein |
| Cold shock protein | *cspB* | 918, 1411, 3157 | COG1278 | cold shock protein |
|  | *cspA* | 1367 | COG1278 | cold shock protein |
|  | *ydjO* | 2208 | No | cold shock protein |
|  | *cspLA* | 1656, 2656, 2659, 3376, 3378, 3559, 3741 | COG1278 | cold shock protein |
| Heat shock protein | *yabO* | 54 | COG1188 | heat shock protein |
|  | *hspc4-1* | 1880, 2903 | COG0071 | heat shock protein |
| Alkaline shock protein | *asp23* | 2517 | COG1302 | alkaline-shock protein |
| Rhodanese | *glpE* | 4433 | COG0607 | rhodanese-like domain-containing protein |
|  | *yrkF* | 3305, 4794 | COG0607 | rhodanese-like domain-containing protein |
| Amino acid decarboxylase | *speA* | 23, 1297 | COG1982 | arginine decarboxylase |
|  | *panD* | 1336 | COG0853 | aspartate 1-decarboxylase |
| Urease | *ureI* | 2749 | — | urease accessory protein |
|  | *ureH* | 2926 | COG2215 | urease accessory protein |
|  | *ureD* | 2927 | COG0829 | urease accessory protein |
|  | *ureG* | 2928 | COG0378 | urease accessory protein |
|  | *ureF* | 2929 | COG0830 | urease accessory protein |
|  | *ureE* | 2930 | COG2371 | urease accessory protein |
|  | *ureC* | 2931 | COG0804 | urease subunit |
|  | *ureB* | 2932 | COG0832 | urease subunit |
|  | *ureA* | 2933 | COG0831 | urease subunit |
| Cation/proton antiporters | *mrpA* | 4863 | COG1009 | Na^+^/H^+^ antiporter subunit |
|  | *mrpB* | 4864 | COG2111 | Na^+^/H^+^ antiporter subunit |
|  | *mrpC* | 4865 | COG1006 | Na^+^/H^+^ antiporter subunit |
|  | *mrpD* | 4866 | COG0651 | Na^+^/H^+^ antiporter subunit |
|  | *mrpE* | 4867 | COG1863 | Na^+^/H^+^ antiporter subunit |
|  | *mrpF* | 4868 | COG2212 | Na^+^/H^+^ antiporter subunit |
|  | *mrpG* | 4869 | COG1320 | Na^+^/H^+^ antiporter subunit |
|  | *nhaC* | 2143 | COG1757 | Na^+^/H^+^ antiporter subunit |
|  | *mleN* | 1207 | COG1757 | Na^+^/H^+^ antiporter subunit |
|  | *nhaK* | 2798 | COG0025 | Na^+^/H^+^ antiporter subunit |
|  | *nhaP2* | 1896 | COG3263 | K^+^/H^+^ antiporter |

Note: Genes were annotated based on Non-redundant (Nr) protein database (a: COG database; b: Swissprot database; c: Pfam database)


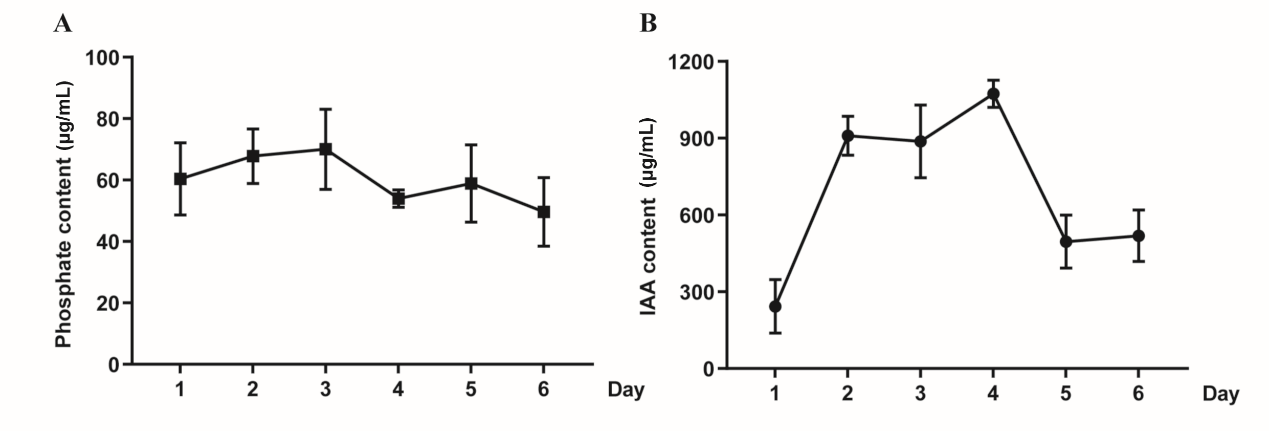


**FIGURE S1│** IAA-producing and phosphate-solubilizing capacity of HGS7 strain over time. **(A)** phosphate-solubilizing ability; **(B)** IAA-producing ability. Data represent the mean ± the standard deviation (n = 3).


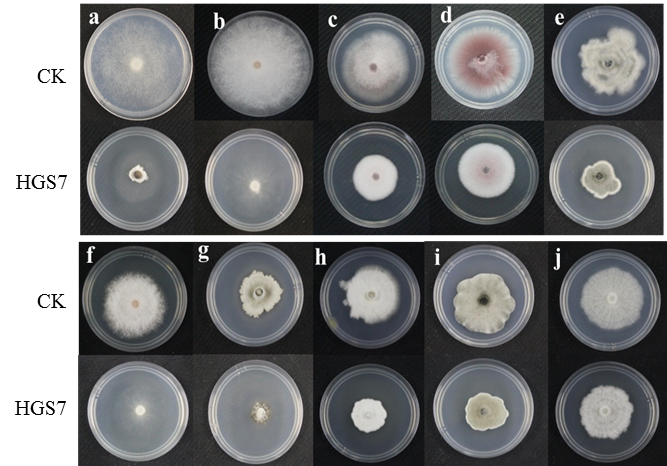


**FIGURE S2**│The inhibitory activities of cell-free supernatant obtained from *B. megaterium* HGS7 on different plant pathogens. Growth inhibition was calculated after 3 days of inoculation. *Sclerotinia sclerotiorum*, *Phoma exigua*, and *Scleromitrula shiraian* were calculated after 1, 7, and 9 days, respectively. a, *Sclerotinia sclerotiorum*; b, *Botrytis cinerea*; c, *Alternaria alternata*; d, *Fusarium solani*; e, *Phoma exigua*; f, *Fusarium oxysporum*; g, *Scleromitrula shiraiana*; h, *Beauveria bassiana*; I, *Cochliobolus sativus*; j, *Ceratocystis ulmi.*


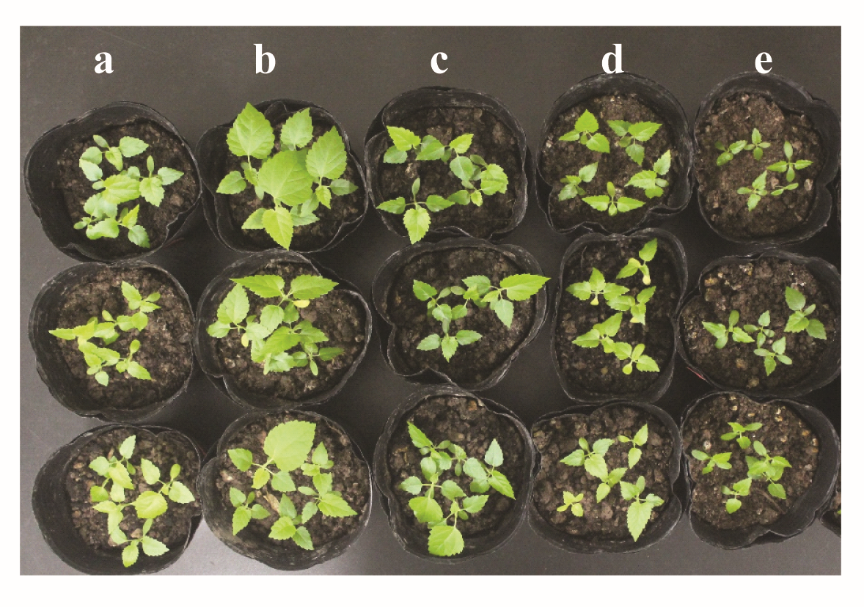


**FIGURE S3**│ Effect of *B. megaterium* HGS7 suspension with different concentrations on the growth of mulberry seedlings observed on 50 days after treatment. a, 1.0 × 10^8^ CFU/mL; b, 1.0 × 10^7^ CFU/mL; c, 1.0 × 10^6^ CFU/mL; d, 1.0 × 10^5^ CFU/mL; e, water.
